# Psychometric assessment of patients with central serous chorioretinopathy and correlation with disease stage and progression: a case control study

**DOI:** 10.1186/s12886-024-03356-2

**Published:** 2024-02-29

**Authors:** Hinrich J. Hufnagel, Claas Lahmann, Hansjürgen Agostini, Clemens Lange, Laurenz J. B. Pauleikhoff, on behalf of the Retina.net CCS study group

**Affiliations:** 1 Eye Center, Medical Center – University of Freiburg, Faculty of Medicine, University of Freiburg, Freiburg, Germany; 2 Department of Psychosomatic Medicine and Psychotherapy, Medical Center – University of Freiburg, Faculty of Medicine, University of Freiburg, Freiburg, Germany; 3 St. Franziskus Eye Center, Münster, Germany; 4 Department of Ophthalmology, University Medical Center Hamburg-Eppendorf, Hamburg, Germany

**Keywords:** Central serous chorioretinopathy, Depression, Anxiety, Stress, Retinal vein occlusion

## Abstract

**Background:**

Central serous chorioretinopathy (CSC) has frequently been associated with increased stress levels as well as an increased prevalence of other psychiatric conditions. This study used standardized psychometric scores to assess stress, depression and anxiety levels of CSC patients and compared them to controls without retinal disease (“healthy”) and with branch retinal vein occlusion (BRVO).

**Methods:**

Monocentric, longitudinal case control study on consecutive CSC patients seen at a tertiary referral center. Controls without retinal disease were recruited from the oculoplastics clinic and those with BRVO from the medical retina clinic. Patients completed pseudonymized tests measuring stress levels (PHQ-stress), depression (PHQ-9) and anxiety (GAD-7) at baseline and at 3- and 6-months follow-up. Higher scores indicated higher trait levels.

**Results:**

65 CSC patients, 19 healthy controls and 19 BRVO patients were included in this study. CSC patients showed significantly higher stress levels at baseline compared to controls (*p* = 0.009), but not compared to BRVO patients (*p* = 1.00). At 3- and 6-months follow-up, no significant difference between groups was observed anymore. Acute CSC patients showed higher scores than those with chronic CSC, which also subsided over time. Depression and anxiety scores did not differ between groups at any timepoint.

**Conclusions:**

Patients with CSC do not show higher initial stress levels than patients with BRVO, while anxiety and depression levels did not differ from controls. Stress may thus rather represent a consequence of the onset of visual deterioration observed in CSC or other ocular diseases.

**Supplementary Information:**

The online version contains supplementary material available at 10.1186/s12886-024-03356-2.

## Introduction

Central serous chorioretinopathy (CSC) is the fourth most common maculopathy, caused by a presumed choroidal dysfunction leading to choroidal hyperpermeability and subsequent accumulation of subretinal fluid (SRF) [1]. This can lead to symptoms such as metamorphopsia, micropsia, hypermetropia, and dyschromatopsia as well as transient or irreversible vision loss due to atrophy of neuronal tissue. The exact aetiology remains unknown [1, 2], but is assumed to be multifactorial with several risk factors being discussed [3, 4].

One of the best established and strongest risk factors is the use of *exogenous* corticosteroids [3, 5–7], regardless of whether they are applied topically or systemically [8, 9]. Higher *endogenous* cortisol levels are also suspected to trigger CSC [5, 10, 11]. As several mental or psychiatric conditions can increase cortisol levels [12, 13], some reports have suggested that they may also trigger CSC. Studies focusing on stress [14–16], depression [14, 15] and anxiety [14, 15, 17, 18] have often shown association with CSC. However, many of these psychological assessments lacked a healthy control group [15, 16] and in particular a control group consisting of patients with another retinal disease to account for a possible influence of visual deterioration on psychometric scores. In addition, patients in these studies were often examined only once, so longitudinal data were missing [14, 16], and validated psychological questionnaires were not used [15].

The Patient Health Questionnaire depression scale (PHQ-9), the Generalised Anxiety Disorder scale (GAD-7) and the stress scale of the Patient Health Questionnaire (PHQ-stress), are among the most validated and widely used psychometric tests for depression, anxiety and stress, respectively [19–21]. Although they have been used in hundreds of research studies and included in numerous clinical practice guidelines, these questionnaires have so far not been used for assessing the psychological state of patients with CSC.

The aim of this study was to assess CSC patients in different stages of the disease using the PHQ-stress, PHQ-9 and GAD-7, measuring stress, depression, and anxiety levels, and compare them to two control groups, one consisting of patients without a retinal pathology and one consisting of patients with branch retinal vein occlusion (BRVO). The same tests were repeated after 3 and 6 months to assess their change over time and their correlation with disease progression.

## Methods

### Participants

Participants were recruited from the outpatient clinics of the Eye Center at the Medical Center of the University of Freiburg, Germany between 01/May/2021 and 30/April/2022. The following three groups were recruited: 1) patients with clinically diagnosed CSC of all stages, 2) participants without retinal or other ocular disease resulting in low vision and 3) patients with branch retinal vein occlusion (BRVO). A total of 103 patients were enrolled in this study, of which 65 patients presented with CSC, 19 patients with BRVO and 19 patients with disease of the ocular adnexa but no retinal disease. Consecutive CSC patients, who were part of the German Retina. Net CSC registry, were included irrespective of their age, sex [22] and best corrected visual acuity (BCVA). The diagnosis of CSC was based on multimodal imaging and included SRF visible on an optical coherence tomography (OCT) scan and, where available, 1 or more regions of active focal leakage combined with RPE window defects visible on fundus fluorescein angiography (FFA), and hyperfluorescent changes on indocyanine green angiography (ICGA) [23–25].

Patients with no current signs or previous history of retinal disease were enrolled as controls. Optical coherence tomography (OCT) was performed to rule out any retinal pathology. To ensure they were not impacted by a reduced visual acuity, participants in this group had to have a BCVA of 20/25 or higher.

To control for the effects of acute macular disease with visual loss similar to CSC, patients with clinically diagnosed retinal vein occlusion were included as a third group in this study. The diagnosis was based on patient records and made by a medical retina consultant based on medical history, fundoscopy, OCT and FFA. To ensure a roughly comparable age to CSC patients, participants from both control groups had to be between 30 and 70 years old on the day of recruitment.

### Clinical examination

All CSC patients underwent slit lamp examination, enhanced-depth imaging optical coherence tomography (EDI-OCT), fundus autofluorescence (FAF), and BCVA measurement at the time of inclusion. Full patient records were screened for which eyes was affected, duration of symptoms and previous treatment in CSC patients, and predefined comorbidities of interest for all patients. These included recent or concurrent use of corticosteroids, psychiatric morbidity (depression, anxiety or eating disorder), arterial hypertension and smoking. Since corticosteroid use was not always recorded for BRVO patients, this was considered not assessable.

### Classification of CSC

CSC patients were initially classified as either acute or chronic CSC based on a recent international consensus [1]. Acute CSC was defined as SRF less than 6 months and no signs of chronicity such as widespread RPE damage or extensive photoreceptor atrophy. Chronic CSC was defined by persistence of SRF for 6 months or longer and signs of chronicity as mentioned above. Patients with a new episode of CSC after a previous complete resolution of SRF were considered acute CSC if they did not show any signs of chronicity. The classification was based on complete multimodal imaging, including OCT, FAF, OCTA, FFA and ICGA imaging, and was performed by one grader (L.P.), who was masked to patient scores on the psychometric assessment tools.

### Psychometric evaluation

Patients were screened using three pseudonymized, self-administered questionnaires on stress (PHQ-stress), depression (PHQ-9), and anxiety (GAD-7). They are specifically designed for their use in primary medicine [26]. The PHQ-stress module consists of 10 items, each of which is rated on a 2-point-scale (0–2), while the PHQ-9 consists of 9 items which are each rated on a 3-point-scale (0–3) and the GAD-7 contains 7 items rated on a 3-point-scale (0–3).

After patients completed the questionnaires, the items of each questionnaire were summed up by one examiner (H.H.) and the resulting overall scores were calculated. A higher score indicates a higher expression of the trait being tested.

### Follow-up examination

All patients were asked to complete the three questionnaires again 3 and 6 months after the initial visit. Data handling and analysis was identical to the baseline visit. For CSC patients that had clinical follow-up examinations after 3- and / or 6-months, multimodal imaging was performed at the clinician’s discretion, which always included OCT. For these patients, the following parameters were assessed on each visit: objective change in SRF based on OCT (increase, stable, decrease) and subjective change in symptoms (better, unchanged, worse).

Change of SRF was assessed by one grader (L.P.) based on available OCT scans. An increase of SRF in one or more OCT scans without a decrease in any of them was graded as “increase”, no change or a redistribution of SRF without a clear change in overall volume was considered as “stable”, while a decrease in all available OCT scans or a clear decrease in total volume was graded as “decrease”. Subjective change in symptoms was based on patient-reported change in visual function as stated in the patient record.

### Data analysis

Pseudonymized psychometric scores and clinical characteristics (age, sex, BCVA) were entered into Microsoft Excel (Microsoft Corp., Redmond, WA, USA). Data was analyzed using R Studio (version 2022.07.2 + 576) [27]. BCVA measured on Snellen charts was converted to logarithm of the minimum angle of resolution (logMAR BCVA) using the formula logMAR BCVA = −log (Snellen fraction) to allow for statistical analysis. Statistical significance was defined as *p* < 0.05. Psychometric scores were analyzed between study groups and for each examination using Kruskal-Wallis test. This was followed by pairwise Mann-Whitney U test with Bonferroni’s adjustment. Missing data was judged to be missing at random.

## Results

### Baseline characteristics

The mean age (±SD) of patients at baseline was similar in all three groups: 53.9 years (±9.7) for CSC patients, 59.1 years (±9.4) for patients without retinal disease and 63.3 years (±6.9) for BRVO patients, respectively. 50 CSC patients (76.9%), 8 patients without retinal diseases (42.1%), and 15 BRVO patients (78.9%) were male. CSC patients showed a higher mean logMAR BCVA than controls without retinal disease (0.14 ± 0.21 ≈ 20/25 Snellen equivalent vs. 0.01 ± 0.02 ≈ 20/20 Snellen equivalent, *p* = 0.008), but a lower mean logMAR BCVA than BRVO patients (0.14 ± 0.21 ≈ 20/25 Snellen equivalent vs. 0.21 ± 0.19 ≈ 20/32 Snellen equivalent, *p* = 0.03). As expected based on previous reports [6, 28], CSC patients showed a higher percentage of steroid therapy (19% vs. 5% in controls), while arterial hypertension was more common in BRVO patients (53% vs. 22% for CSC and 21% for healthy controls). Anxiety disorders were more prevalent in BRVO than in the two other groups (16% vs. 8% for CSC patients and 5% for healthy controls) and depression varied between 11% (healthy controls) and 21% (BRVO). Of note, CSC patients did not show more psychiatric comorbidities than the other two groups. Full baseline characteristics can be found in Table 1.

**Table 1:** Baseline characteristics of the patients included in this study ^a,b^

	CSC *n* = 65	BRVO *n* = 19	No retinal pathology *n* = 19
**Age (yrs) ± SD**	53.9 ± 9.7	63.3 ± 6.9	59.1 ± 9.4
**Male (%)**	50 (77%)	15 (79%)	8 (42%)
**Female (%)**	15 (23%)	4 (21%)	11 (58%)
**Right eye (%)**	21 (32%)	8 (42%)	–
**Left eye (%)**	25 (39%)	8 (42%)	–
**Both eyes (%)**	19 (29%)	3 (16%)	–
**Baseline logMAR ± SD**	0.14 ± 0.21	0.21 ± 0.19	0.01 ± 0.02
**Comorbidities**			
**Steroid therapy (%)**	12 (19%)	–	1 (5%)
**Anxiety disorder (%)**	5 (8%)	3 (16%)	1 (5%)
**Depression (%)**	8 (12%)	4 (21%)	2 (11%)
**Eating disorder (%)**	0 (0%)	1 (5%)	1 (5%)
**Arterial hypertension (%)**	14 (22%)	10 (53%)	4 (21%)
**Smoker (%)**	4 (6%)	2 (11%)	1 (5%)

^a^Continuous variables are reported as mean ± standard deviation (SD)

^b^Categorical variables are reported as number (percent)

### Psychometric scores at baseline

**Fig. 1:**
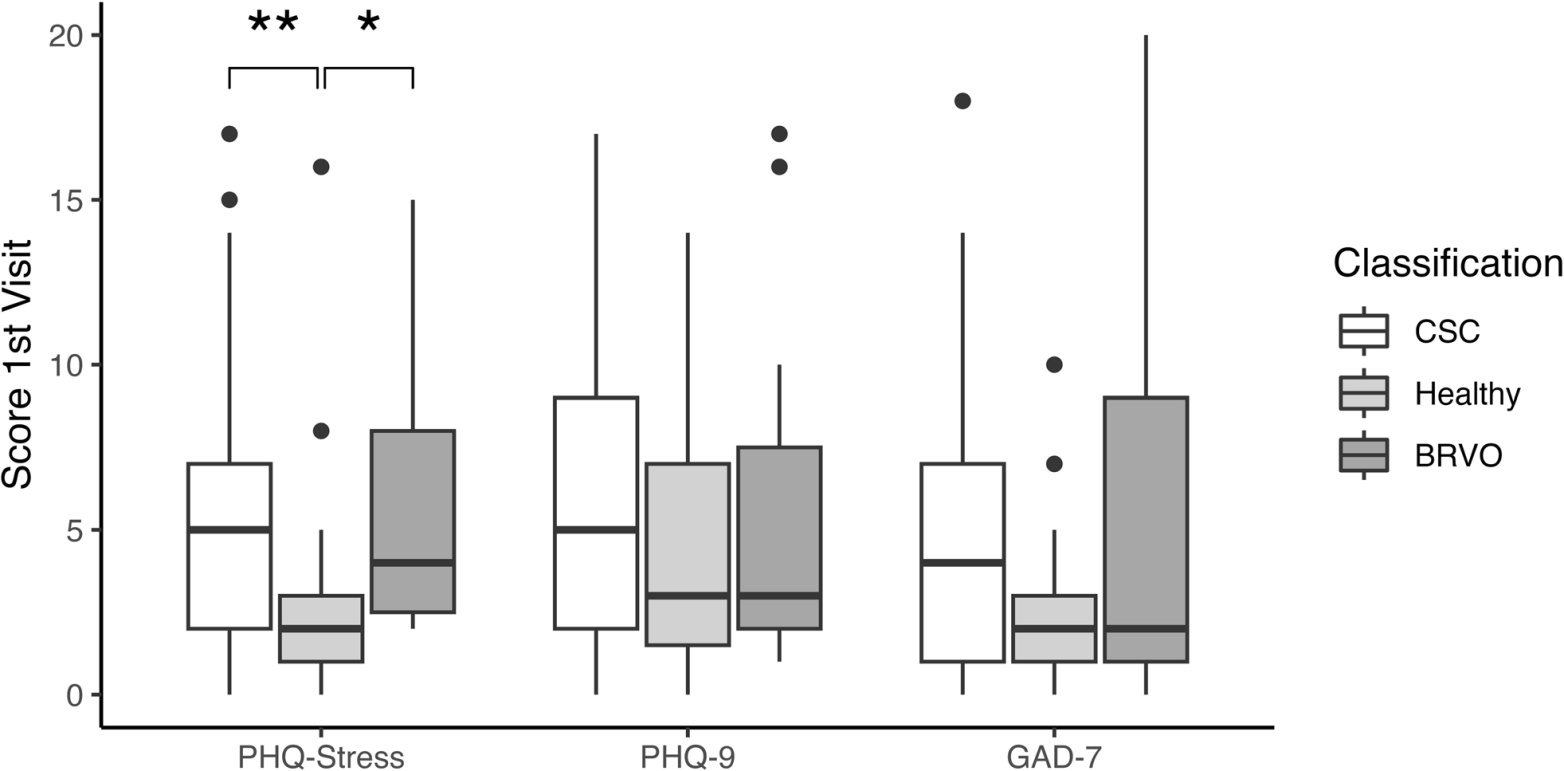
Baseline psychometric scores for all groups. Boxplots of PHQ-stress, PHQ-9 (measures depression) and GAD-7 (measures anxiety) scores for the three groups: central serous chorioretinopathy (CSC), branch retinal vein occlusion (BRVO) and no retinal pathology (“healthy”). One asterisk indicates a *p*-value of < 0.05, two asterisks a p-value of < 0.01

Patients with CSC showed significantly higher stress scores (PHQ-stress) (median [IQR]) at baseline than patients without retinal diseases (5 [2-7] vs. 2 [1-3], *p* = 0.009, Fig. 1). However, patients with BRVO also revealed significantly higher initial stress scores compared to controls without retinal disease (4 [2.5–8] vs. 2 [1–3], *p* = 0.013) and no significant difference in stress scores was observed between CSC and BRVO patients (5 [2–7] vs. 4 [2.5–8], *p* = 1.00).

Mean depression score (PHQ-9) at baseline showed no significant difference between CSC patients compared to healthy controls (5 [2–9] vs. 3 [1.5–7], *p* = 0.71) or BRVO patients (5 [2–9] vs. 3 [2–7.5], *p* = 1.00). Similarly, anxiety levels (GAD-7) were also not significantly different between CSC patients and healthy controls (4 [1–7] vs. 2 [1–3], *p* = 0.28), or BRVO patients (4 [1–7] vs. 2 [1–9], *p* = 1.00).

### Psychometric scores at follow-ups

The same questionnaires were repeated at the first follow-up 3 months after baseline and were completed by 43 CSC patients, 16 controls, and 16 BRVO patients; the second follow-up 6 months after baseline was completed by 57 CSC patients, 14 controls, and 15 BRVO patients.

Stress, depression and anxiety scores were not statistically significant between any of the groups for any of the follow-ups (data not shown).

### Differences between CSC subgroups

To further investigate differences in stress levels and account for a possible impact of chronicity, CSC patients were classified into acute (*n* = 26, 40%) and chronic CSC (*n* = 39, 60%) based on their clinical presentation at baseline. Acute CSC patients showed a lower mean logMAR BCVA than chronic CSC patients (0.07 ± 0.13 ≈ 20/25 Snellen equivalent vs. 0.19 ± 0.25 ≈ 20/32 Snellen equivalent, *p* = 0.004). As expected, mean duration of symptoms was shorter for acute CSC patients compared to chronic ones (3.1 ± 1.9 vs. 73.7 ± 72.7 months, *p* < 0.001). Acute patients had a minimum duration of symptoms of 0.2 months and a maximum of 5.9 months. Chronic ones had a minimum duration of symptoms of 7.3 months and a maximum of 307.9 months. Patient characteristics for both groups are shown in Table 2.

**Table 2:** Comparison of baseline characteristics of patients with acute and chronic central serous chorioretinopathy ^a,b^

	Acute CSC *n* = 26	Chronic CSC *n* = 39
**Baseline logMAR ± SD**	0.07 ± 0.13	0.19 ± 0.25
**Mean duration of symptoms (months) ± SD**	3.1 ± 1.9	73.7 ± 72.7
**Minimum (months)**	0.2	7.3
**Maximum (months)**	5.9	307.9
**Previous treatment**		
**Eplerenon**	2 (7.7%)	6 (15.4%)
**Acetazolamide**	2 (7.7%)	6 (15.4%)
**NSAID eyedrops**	1 (3.9%)	4 (10.3%)
**Argon laser**	2 (7.7%)	5 (12.8%)
**Photodynamic therapy (PDT)**	0 (0.0%)	8 (20.5%)
**Anti-VEGF**	2 (7.7%)	14 (36%)

^a^Mean ± standard deviation (SD)

^b^Number (percent)

**Fig. 2:**
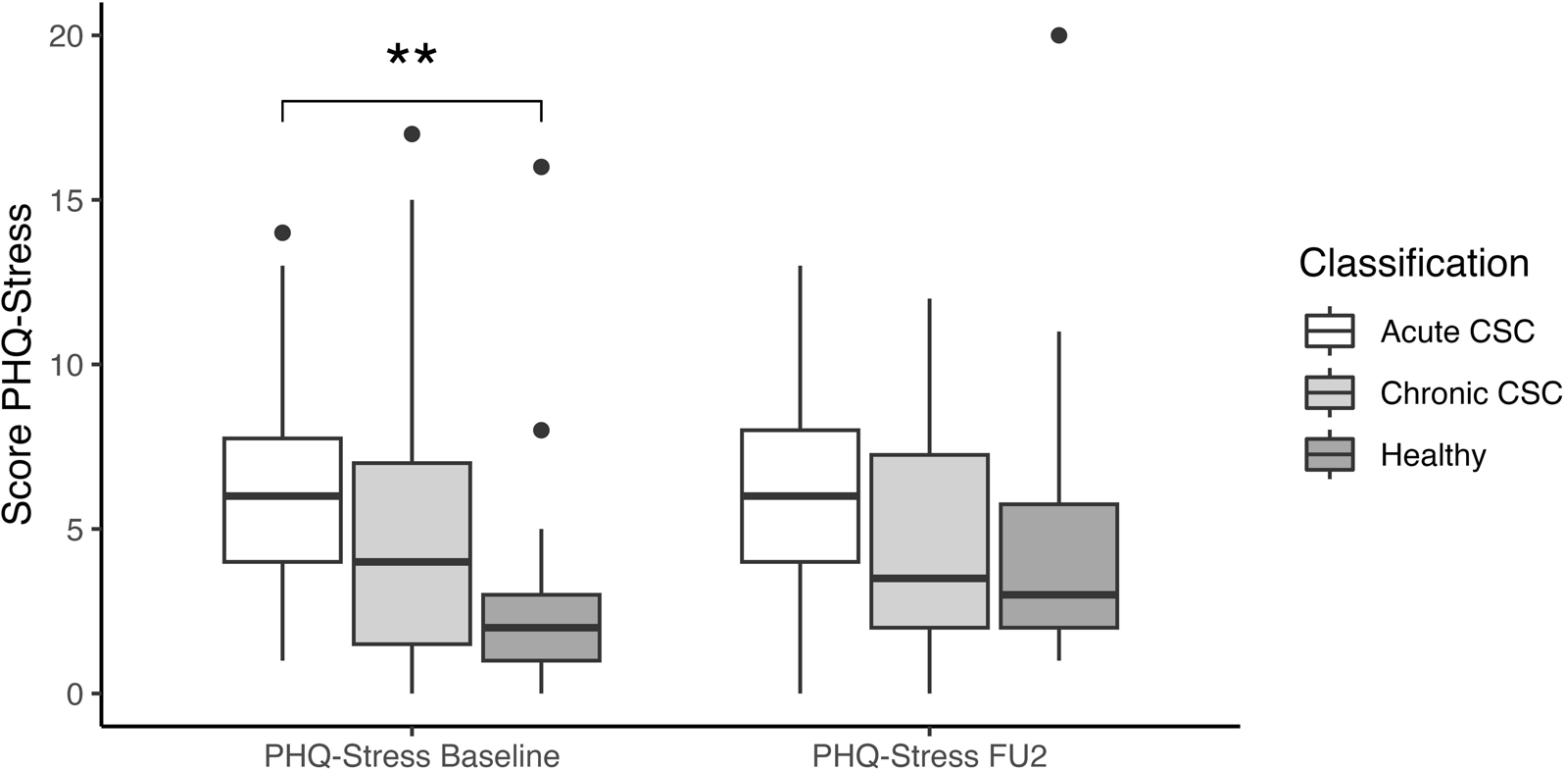
Stress scores at baseline and second follow-up for acute and chronic central serous chorioretinopathy compared to controls. Boxplots showing PHQ-stress scores for acute and chronic CSC patients compared to controls without a retinal pathology. Two asterisks indicate a p-value of < 0.01

Acute CSC patients showed higher stress scores at baseline compared to healthy controls (6 [4–7.75] vs. 2 [1–3], *p* = 0.001, Fig. 2), while chronic CSC patients showed no significant difference compared to healthy controls (4 [1.5–7] vs. 2 [1–3], *p* = 0.137). The difference between acute and chronic CSC patients was also not statistically significant (6 [4–7.75] vs. 4 [1.5–7], *p* = 0.091). At final follow-up, however, all three groups showed no significant differences anymore (acute CSC 6 [4–8], chronic CSC 3.5 [2–7.25], healthy controls 3 [2–5.75], all *p*-values > 0.05).

### Impact on patient prognosis

**Fig. 3:**
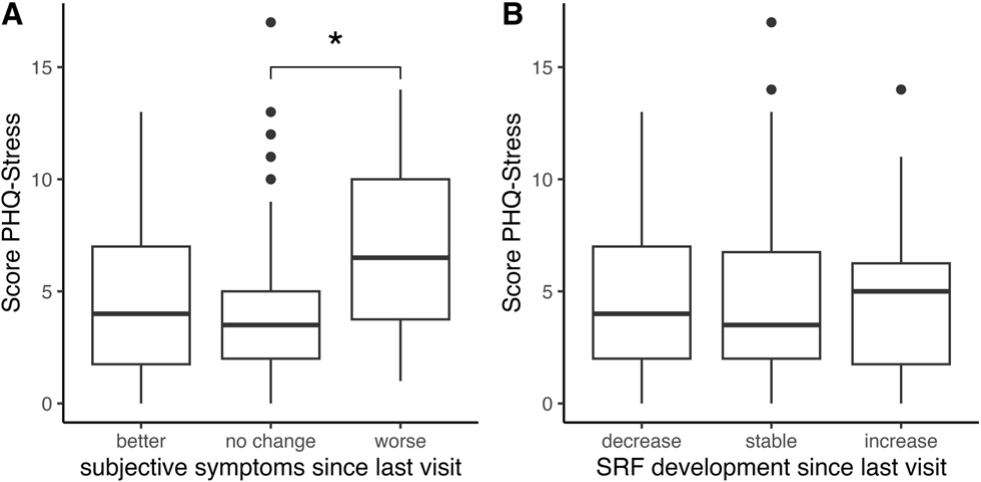
Correlation between stress scores and subjective and objective changes in subretinal fluid during follow-up. One asterisk indicates a p-value of < 0.05

We further examined how the clinical course of CSC patients related to their stress scores. CSC patients were therefore divided into three groups depending on either their subjective change in symptoms since their last visit (Fig. 3A) or the objective change in SRF over time (Fig. 3B). CSC patients who subjectively felt their symptoms worsened since their last visit showed significantly higher stress scores compared to those with no change in symptoms (6.5 [3.75–10] vs. 3.5 [2–5], *p* = 0.028), while there were no significant differences between the other subgroups. When looking at objectively measured fluid on OCT, however, we did not observe a statistically significant difference in stress scores between patients with increased, stable, or decreased SRF (5 [1.75–6.25] vs. 3.5 [2–6.75] vs. 4 [2–7], all *p*-values > 0.5).

## Discussion

Our study shows that CSC patients have higher baseline stress levels compared to healthy controls, but not compared to BRVO patients. During follow-up, this difference in stress levels subsided. We also found no difference in depression or anxiety scores between the groups and no correlation of these scores with objective changes in SRF.

Psychological traits of CSC patients have been a matter of debate among ophthalmologists for decades. As early as 1987, Yannuzzi hypothesized that individuals with a “type A personality” are at higher risk of developing CSC [29]. This term, established by Friedman and Rosenman, describes achievement-oriented, competitive, fast-paced, and impatient individuals, who were reported to be at a much higher risk of developing cardiovascular diseases [30]. Since Type A behavior has been associated with higher stress levels and increased endogenous cortisol levels [31], this seems plausible considering the strong association of CSC to exogenous steroid use. In his study, Yannuzzi showed that more CSC patients exhibited type A behavior than patients with other chorioretinal diseases or non-chorioretinal ocular conditions, which are similar control groups to the ones used in our study [29]. Since these results were published, the classification of individuals into personality types has been criticized and mostly been replaced by multidimensional models [32]. In our study, we therefore focused on a direct assessment of stress, depression, and anxiety levels rather than underlying personality types. Our data does, however, not show significant differences between the three groups on either stress levels, anxiety or depression scores at the 3- and 6-months follow-up visits. This could indicate that psychological differences between groups are an “acute”, short-term phenomenon rather than long-standing differences between patient’s personalities.

Most studies of individual psychological traits (rather than just personality types) compared CSC patients with healthy age- and sex-matched controls [14, 17], if they had control groups at all [15, 16, 18]. Consistent with these reports [14–16], we also found higher stress level in CSC patients compared to a control group without retinal disease (Fig. 1). When compared to BRVO patients, however, we did not observe a statistically significant difference in stress levels. Moreover, BRVO patients themselves showed a statistically significant increase in stress levels compared to healthy controls, even though BRVO is not considered to be “triggered” by stress. BRVO patients had lower visual acuities than CSC patients though. Since a low vision-related quality of life in BRVO patients has been reported [33], it seems possible that vision loss itself can cause higher stress levels and other psychosomatic symptoms. This highlights the difficulty of differentiating whether stress is a *cause* or a *consequence* of vision loss [34]. Based on our results, it seems plausible that increasing stress levels is a *consequence* rather than a *cause* of CSC. This could also explain why we found a correlation between stress scores and subjective change in symptoms, while no correlation with objective change in SRF was observed (Fig. 3). Because of the difficulty in separating cause and consequence, conclusions drawn on studies without a control group of vision-impaired patients should be interpreted with care.

Our study also observed a difference in stress levels between acute and chronic CSC patients. While acute CSC patients showed higher stress levels compared to healthy patients, this was not true for chronic CSC patients (Fig. 2). On the follow-up visits, however, this difference disappeared. This could again be explained by stress as a consequence of visual deterioration rather than its cause. It has also been reported that acute CSC patients show more psychosomatic symptoms and less favorable coping than chronic patients [14, 35], as chronic cases may be more used to their condition and have found a way to deal with the disease. Moreover, they seek more social support than acute CSC patients [36]. Even though it does not trigger the disease, psychological counselling and stress reduction may therefore still play a role in managing symptomatic CSC patients [34].

In contrast to previous publications, we did not find evidence for higher levels of depression [14, 15] and anxiety [14, 15, 17, 18] in CSC patients compared to our control groups (Fig. 1). We also did not find a higher patient-reported incidence of psychiatric diseases in CSC patients compared to controls (Table 1). Even though underreporting of these diseases cannot be excluded, we do not believe this severely impacted the results, since it was assessed as part of the pseudonymized questionnaires. The rationale of how depression and anxiety may impact CSC also seems questionable, since they do not necessarily impact endogenous cortisol levels in individuals [12, 37]. Due to the methodological challenges in previous studies mentioned above, it can currently not be concluded that CSC patients in general are at a higher risk of psychiatric comorbidities.

We acknowledge that our study has some limitations including the relatively smaller cohort sizes of controls without visual impairment and BRVO patients, which could not be completely matched to the baseline characteristics of CSC patients due to the difference in patient demographics of these diseases. Since this study was performed at a tertiary referral center, CSC patients overall may have had a longer duration of symptoms than in a typical secondary care setting. Thus, we were not able to include patients at the moment when CSC occurred and assess their psychosomatic just then or before. However, we recruited patients at a very early stage of the disease. By recruiting consecutive CSC patients and repeating the psychometric tests at two follow-up visits, we nevertheless believe our results are valid and can help to settle the long-standing debate on psychological characteristics of CSC patients.

In conclusion, our study shows that psychological stress may rather be a consequence than a cause of CSC. We also did not observe higher depression or anxiety scores or more prevalent psychiatric comorbidities in CSC patients compared to controls. At the same time, patient with any disease suffering from psychological stress may be counselled on stress reduction to best address patient needs.

### Supplementary Information

**Supplementary Material 1.**

## Data Availability

The datasets used and/or analysed during the current study are available from the corresponding author on reasonable request.

## Abbreviations

BCVA: Best corrected visual acuity
BRVO: Branch retinal vein occlusion
CSC: Central serous chorioretinopathy
EDI-OCT: Enhanced Depth Imaging OCT
FAF: Fundus autofluorescence
FFA: Fundus fluorescein angiography
GAD: Generalised Anxiety Disorder Scale
ICGA: Indocyanine green angiography
IQR: Interquartile range
OCT: Optical coherence tomography
PHQ: Patient Health Questionnaire
RPE: Retinal pigment epithelium
SD: Standard deviation
SRF: Subretinal fluid
