# Supplementary material for: Psychometric assessment of patients with central serous chorioretinopathy and correlation with disease stage and progression: a case control study
Source: BMC Ophthalmol. 2024 Feb 29;24:92. doi: 10.1186/s12886-024-03356-2 (PMC10902987; doi:10.1186/s12886-024-03356-2)
Supplement: Supplementary file 1 — Supplementary Material 1. [file 12886_2024_3356_MOESM1_ESM.docx]

| **Diagnosis** | **Patients without retinal disease**  **n = 19** |
| --- | --- |
| Dermatochalasis | 5 (26%) |
| Ptosis | 5 (26%) |
| Basalioma | 3 (16%) |
| Papilloma | 3 (16%) |
| Epidermal cyst | 2 (11%) |
| Postsaccal stenosis of lacrimal duct | 2 (11%) |
| Trichiasis | 2 (11%) |
| Entropion | 1 (5%) |
| Eyelid nevus | 1 (5%) |
| Cyst of Moll | 1 (5%) |

**Supplemental table 1. Overview of diagnoses of patients without retinal disease**
